# Treatment of an Avulsed and Ankylosed Incisor through Single Tooth Alveolar Osteotomy and Conventional Orthodontic Mechanisms

**DOI:** 10.3390/children9050732

**Published:** 2022-05-17

**Authors:** Georgios Vasoglou, Chrysi Christina Markomanolaki, Michail Vasoglou, Andreas Markomanolakis

**Affiliations:** 1 Department of Orthodontics, 401 General Military Hospital of Athens, 138 Mesogeion Avenue, 11525 Athens, Greece; gio.vasoglou@gmail.com; 2 Private Orthodontic Practice, Ahilleos Kirou 74, 11525 Athens, Greece; chrismarkdent@yahoo.gr (C.C.M.); a.markomanolakis@gmail.com (A.M.); 3 Department of Orthodontics, School of Dentistry, National and Kapodistrian University of Athens, 2 Thivon Str, Goudi, 11527 Athens, Greece

**Keywords:** tooth ankylosis, dental trauma, osteotomy, distraction osteogenesis

## Abstract

We present the treatment of an injured and avulsed incisor (which was afterwards ankylosed), by subapical osteotomy and conventional orthodontic mechanisms. A 9-year-old boy presented for orthodontic treatment with an injured and avulsed central incisor, which, after initial repositioning, ended up with ankylosis and severe root resorption. The case was treated by single tooth alveolar osteotomy and distraction osteogenesis through conventional orthodontics, advocating for the floating bone concept due to the short vertical distance by which the tooth alveolus part had to be relocated. Orthodontic treatment of the avulsed central incisor was completed after osteotomy and distraction osteogenesis, and the tooth was restored to its proper position, aiming to address issues of aesthetics and function for the time being. The patient was finally referred to a prosthodontist for future and final implant rehabilitation due to severe root resorption. Distraction osteogenesis after surgical procedures is a reliable solution for dealing with an ankylosed and submerged tooth.

## 1. Introduction

Dental trauma is common in children and adolescents. It is considered to be associated with class II malocclusion, increased overjet, protrusive teeth, lip incompetence and mouth breathing [1,2]. An injury displacement of an incisor, which compromises the integrity of the periodontal ligament, often leads to ankylosis [3]. Ankylosis is also the result after avulsion and repositioning an injured incisor. This situation is characterized by the fusion of cementum or dentin with the alveolar bone and is clinically evidenced by a submerged tooth and gingival discrepancies as the surrounding structures continue their vertical growth [4]. Compromised aesthetics demand an effective treatment for the young patient immediately after the incident, but also future reliable rehabilitation.

Treatment options are related to the general condition and orthodontic situation. Extraction of the ankylosed tooth and prosthetic rehabilitation, extraction and orthodontic space closure if a crowding exists, and surgical luxation (if the ankylosis is at a certain point only) in order to set the tooth free and orthodontically restore it to its original position [5], are some treatment options. Decoronation has also been proposed in order to preserve the bone for future prosthetic rehabilitation with an implant [6].

A surgical approach through a subapical osteotomy and subsequent distraction osteogenesis is an option supported by some clinicians. It aims at new bone formation, as the movable alveolus part with the tooth is guided to its new position, with a certain amount of movement in time [7,8,9,10,11,12].

The purpose of this paper is to present a case report of the treatment of an injured and avulsed incisor. The tooth was repositioned to its proper place but then was ankylosed. Subapical osteotomy and orthodontic repositioning of the tooth alveolus part was advocated. The aim of treatment was to relocate the tooth for aesthetic reasons during adolescence and early manhood and to preserve the integrity of the alveolar process for future implant rehabilitation as the tooth later presented a severe root resorption.

## 2. Materials and Methods

**Figure 1:**
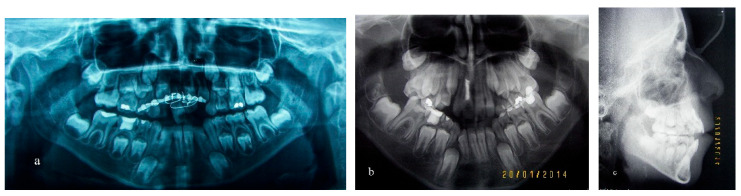
Radiographic examination. (**a**) Panoramic radiograph after repositioning and splinting, (**b**) panoramic radiograph after endodontic treatment, (**c**) lateral cephalogram before orthodontic treatment.

A 9-year-old boy had an accident while playing with his peers, and the upper right incisor was injured and avulsed. The tooth was stored in a cup filled with milk, and it was easily and immediately found. Repositioning of the tooth took place 8 h after the accident, in the oral and maxillofacial department of a hospital, and a surgical splint was fitted (Figure 1a).

**Figure 2:**
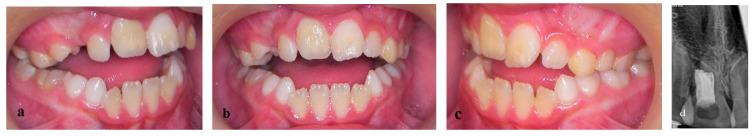
Initial photographs before orthodontic treatment. (**a**–**c**) Patient presented a severe open bite, (**d**) periapical radiograph on which root resorption of 11 is evident.

The splint was removed after 15 days and pulp vitality was tested. Since the tooth was non-vital, a root canal treatment was conducted (Figure 1b). The patient was then referred for orthodontic examination in a private orthodontic office. After an observation period of several months, clinical and radiographic evaluation revealed an open bite in the lateral and front segment due to a skeletally narrow upper jaw, divergent upper and lower jaw levels (Figure 1c) and a tongue thrust (Figure 2a–c). Root resorption of the right incisor was also evident in a periapical radiograph (Figure 2d).

**Figure 3:**
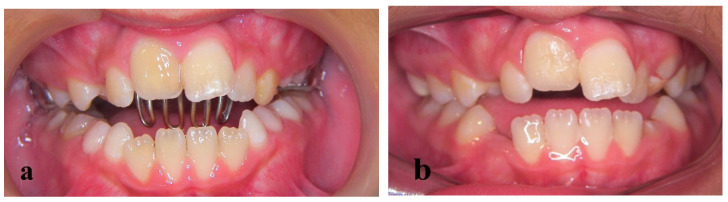
Functional treatment by a tongue crib. (**a**) Tongue crib appliance in place, (**b**) improvement in the open bite and first sign of ankylosis of right central incisor.

A functional treatment by a tongue crib (Figure 3a) was decided and the injured incisor was monitored clinically and radiographically. Ten months later, a significant improvement in the open bite was evident but so was ankylosis of the right incisor. The tooth presented a lack of mobility, seemed submerged (Figure 3b), and a metallic sound on percussion was noted. These symptoms, in conjunction with the lack of periodontal space that was already noted in a previous periapical radiograph (Figure 2d), confirmed the ankylosis.

Detailed orthodontic treatment with fixed appliances began at the age of twelve. The aim was to deal with crowding and skeletal problems and finally reposition the ankylosed tooth by subapical osteotomy, moving the alveolus part with the incisor to its correct position. This approach was recommended in order to resolve aesthetics for the moment and preserve the alveolar process dimensions and bone quality for future implant rehabilitation.

**Figure 4:**
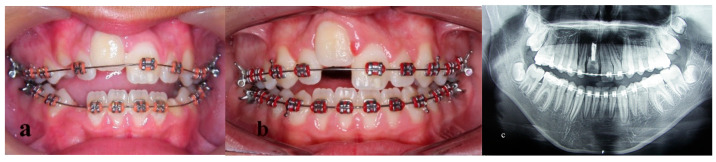
Treatment progress photographs during fixed appliance treatment. (**a**) Four months after treatment, (**b**) 12 months after treatment, (**c**) panoramic radiograph presenting adequate space for osteotomy.

Fixed (0.022 inch-slot) edgewise appliances were fitted in the upper and lower arch. The right central incisor was left untouched for the moment. After leveling and alignment with elastomeric wires (Figure 4a,b), stainless-steel rectangular wires, with an open-coil spring between 12 and 21 teeth, were used to create adequate space for an interdental and subapical osteotomy. A new panoramic radiograph confirmed the situation (Figure 4c).

**Figure 5:**
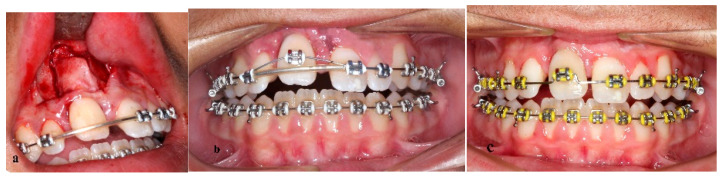
Surgical procedure. (**a**) Subapical osteotomy, (**b**) 0.014 inch NiTi segmented arch wire for vertical distraction, (**c**) ankylosed incisor almost in place.

A full-thickness mucoperiosteal flap was reflected under local anesthesia, and two vertical and a horizontal subapical osteotomy were performed (Figure 5a). Mobility of the alveolus part was confirmed and care was taken to preserve the blood supply through contact of the palatal mucosa with the movable part of the alveolar bone. Then, the flap was repositioned and sutured. Seven days after surgery, a bracket was placed on the incisor and a 0.014 inch NiTi segmented arch wire from the right canine to the left incisor was fitted, while a baseline stainless-steel 0.016 × 0.022 inch arch wire was in place (Figure 5b). The patient was examined every two days for progress monitoring.

## 3. Results

**Figure 6:**
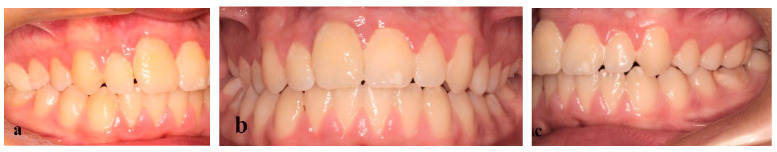
Intraoral final photographs. (**a**–**c**) Ankylosed incisor in the correct place and a small gingival discrepancy.

The movable alveolus part with the ankylosed incisor was nearly restored to its proper position after 12 days (Figure 5c). Therapy continued with a 0.016 × 0.022 inch NiTi continuous arch wire and was completed with space closure and conventional orthodontic procedures. The consolidation phase lasted 6 weeks with a 0.019 × 0.025 inch SS rectangular arch wire and then the appliances were removed (Figure 6a–c).

**Figure 7:**
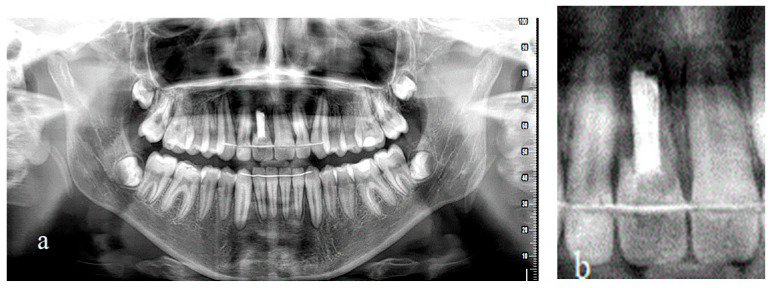
(**a**) Final panoramic radiograph. Fixed retainers for upper and lower arch. Bone healing around 11 was completed. (**b**) Magnified detail from the adjacent panoramic radiograph, presenting severe root resorption of 11 and unfortunately apical root resorption of 12 and 21.

Retention to the upper and lower arch was delivered through a fixed retainer (Figure 7). Removable retainers were also provided.

## 4. Discussion

Ankylosis of a traumatized and displaced tooth is a common incident. It can be avoided by early tooth movement, according to some researchers [13,14,15].

Observation for more than 3 weeks can be risky. Therefore, when trauma and displacement of the anterior teeth occur, the first concern of the clinician is to move the tooth to its original position and splint it with neighboring teeth to allow the healing process of the pulp and periodontal ligament [16].

Orthodontic treatment, if the relocation of the injured tooth by hand is impossible, is then to be considered [17]. Light force delivered through super-elastic wires can be effective in repositioning the tooth and thus monitoring for possible pulp necrosis. In this case, endodontic access and treatment will be easier and more effective [18].

The time of orthodontic treatment after trauma for vital teeth is questionable. Kindelan et al. [19] suggest 3 months of waiting before orthodontic treatment for minor injuries, and 6 months to 1 year for more severe injuries. Nevertheless, root resorption is a potential result in endodontically and afterwards orthodontically treated teeth [20]. Hunter et al. [21] found an equal risk of root resorption between endodontically treated and vital teeth regarding orthodontic treatment, while Remington et al. [22] and Spurrier et al. [23] reported a reduced risk for endodontically treated teeth. Study evidence leads to a nonsignificant difference with regard to root resorption, if the same ordinary orthodontic forces are applied to endodontically treated and vital teeth, assuming that the root therapy is sufficient [24].

On the other hand, when injury results in avulsion, the tooth is recommended to be repositioned in a short time, as a possible long delay may be dangerous [25]. Extraoral dry time is of great importance for a successful replantation, as, after 30–60 min, the periodontal ligament cells are irreversibly damaged. In order to avoid such an incident, storage of the avulsed tooth, for a short time till replantation, in an isotonic solution is suggested. Milk, which was used in this case, is the most recommended medium [26], but Hank’s balanced salt solution, propolis, oral rehydration salts, rice water and cling film have been proven to demonstrate efficacy at preserving the PDL cell viability [27]. Unfortunately, there is often a delay between the avulsion and relocation of the tooth. A surgical splint should stabilize the tooth, or splinting should be considered by placing brackets and an orthodontic wire. Endodontic therapy for nonvital teeth must follow splinting. Afterwards, ankylosis is likely to happen and root resorption is a possibility [28]. Since such accidents happen at a young age, long before orthodontic treatment usually starts for other reasons, monitoring the tooth for some time is often recommended. It is suggested that when endodontic therapy has been carried out after trauma and pulp necrosis, a one-year period of monitoring the tooth before orthodontic treatment is recommended to ensure complete healing, possible root resorption and the absence of ankylosis [29].

The incident of ankylosis and thus an abnormal ridge development in avulsed and replanted teeth led to the suggestion of not replanting at all [30,31]. In addition, decoronation is a procedure in which only the root of a replanted tooth is preserved, when ankylosis is evident, in order for the alveolar ridge to develop normally, in a growing patient [32,33].

In our case, since trauma and avulsion took place at the age of nine, a decision was made to monitor the tooth after repositioning and splinting, for some time clinically and radiographically, for ankylosis and possible root resorption. Unfortunately, not only ankylosis but severe root resorption was confirmed. Orthodontic treatment was scheduled under the condition that the ankylosed incisor would not be involved until the last phase, when surgical reposition with a subapical osteotomy would be attempted. Our goal was to preserve the tooth even if resorption was evident for bone integrity and future implant rehabilitation. This is proposed to be delayed until skeletal growth is completed. Skeletal maturation for implant therapy can be addressed by comparing a conventional radiograph of the hand and wrist against a standardized atlas [34]. However, an arrest in skeletal growth cannot be used as the single measure for the appropriate time of implantation. Eruption of neighboring teeth imposes a risk of implant infraposition, so it is highly recommended that any orthodontic treatment before implant therapy in young individuals should aim at establishing ideal contact between upper and lower incisors [35].

Distraction osteogenesis after subapical osteotomy has been proposed as a procedure to enhance alveolar ridge integrity [36]. This procedure permits the proliferation of soft tissues along with bone expansion and should be preferred when the overall movement is significant. It is preferable to extraction or even decoronation because a provisional prosthesis is avoided.

Several approaches to performing distraction osteogenesis have been proposed. Isaacson et al. [9] applied distraction orthodontics to an ankylosed incisor, after subapical osteotomy, through a 1 mm step on a stainless-steel arch wire every 2 weeks. Kinzinger et al. [37] used a bone-borne distraction system for vertical incisor repositioning. The rate of activation was 0.6 mm/day (8 days of distraction) and final repositioning (after distractor removal) was achieved by the floating bone concept with a segmented super-elastic arch wire. Alcan [10] used a miniature tooth-borne distractor (MTD) and certain bends in the arch wire.

Kim et al. [38] used a pre-expanded screw as a tooth-borne distractor and activated it 1 mm per day for the first 4 days and 0.4 mm per day for the remaining days, until an overcorrection of 1 mm was achieved, to account for the continued vertical growth of the dentition.

Doshi et al. [39] used a hyrax screw for the vertical repositioning of a lateral and a central incisor and the floating bone concept for fine adjustments. Senisik et al. [40] performed vertical alveolar distraction osteogenesis through intraoral elastics from the opposite arch to a mini screw placed in the center of the supporting bone of the ankylosed tooth.

All the above-mentioned techniques involve bulky structures that cause discomfort to young patients. Dolanmaz et al. [41] used continuous distraction forces applied to an ankylosed incisor by super-elastic wires, and in two weeks’ time, the tooth was restored to its proper position. Box elastics were also used finally. This means of distraction was chosen due to the short distance between tooth and the occlusal level. The same approach with nickel–titanium arch wires was recommended by Chang et al. [12], because they deliver constant and light forces, which aid in the distraction procedure.

We used the same method in our case since the vertical distance that had to be covered was minimal and so bone regeneration and soft tissue adaptiveness were not jeopardized by the use of constant force.

Special care should be taken as to the appropriate size of the movable osseous part and the contact with the palatal mucosa for good blood supply. In order to ensure this, adjacent teeth were moved apart by an open-coil spring. In this way, interdental cuts were performed with safety and were slightly divergent to the occlusal plane so as to ensure that movement of the bone–tooth complex was undisturbed. An alveolar ridge defect requires the significant stretching of the soft tissue, which is often a limiting factor and creates potential for relapse [42].

Controversy exists about the latency period. A 14-day [9], 7-day [37], 5-day [39,40] or even 4-day [11] latency period has been proposed, and this seems to be based on the operator’s opinion. In our case, a 7-day latency period was selected, as less time might compromise the healing procedure and the formation of the new transitive connective tissue.

As for the appropriate guidance of the bone part, when a significant distance is to be covered, researchers, as mentioned above, have proposed tooth- or bone-borne screws accompanied by elastic traction or even mini-implant anchorage [40]. In our case, the floating bone concept, with super-elastic arch wires, was used due to the short distance and it was implemented successfully.

An overcorrection might be useful due to the remaining vertical growth of the growing patient. Of course, there is no such problem for an adult case.

The activation rate and consolidation period are matters of controversy too. A rate of 1 mm [9], 0.9 mm [11], 0.6 mm [37] and a combination of 1 mm initially and 0.4 mm later [38] per day have been proposed. Different periods—6 weeks [10], 8 weeks [43] or even 12 weeks [11]—were also proposed for consolidation. In our case, a 6-week consolidation period was planned to ensure an undisturbed healing procedure.

## 5. Conclusions

In this case report, the avulsed, replanted and then ankylosed incisor of a growing patient was restored to its original place through subapical osteotomy and distraction osteogenesis. This approach was chosen to deal with the dentoalveolar and soft tissue defect and to improve aesthetics and gingival symmetry for a period before final implant rehabilitation.

The minimal vertical distance that the tooth had to be relocated led us to perform distraction osteogenesis only through super-elastic arch wires and the floating bone concept. The rate of activation, latency and also the consolidation period need to be further standardized through future studies.

## Data Availability

Data available upon reasonable request.
